# A graph neural network-based bearing fault detection method

**DOI:** 10.1038/s41598-023-32369-y

**Published:** 2023-03-31

**Authors:** Lu Xiao, Xiaoxin Yang, Xiaodong Yang

**Affiliations:** 1 China University of Mining and Technology, Xuzhou, 221116 China; 2 Xinjiang Tianchi Energy Co., Ltd., Changji, 831100 China

**Keywords:** Computational biology and bioinformatics, Computer science, Information technology

## Abstract

Bearings are very important components in mechanical equipment, and detecting bearing failures helps ensure healthy operation of mechanical equipment and can prevent catastrophic accidents. Most of the well-established detection methods do not take into account the correlation between signals and are difficult to accurately identify those fault samples that have a low degree of failure. To address this problem, we propose a graph neural network-based bearing fault detection (GNNBFD) method. The method first constructs a graph using the similarity between samples; secondly the constructed graph is fed into a graph neural network (GNN) for feature mapping, and the samples outputted by the GNN network fuse the feature information of their neighbors, which is beneficial to the downstream detection task; then the samples mapped by the GNN network are fed into base detector for fault detection; finally, the results determined by the integrated base detector algorithm are determined, and the top *n* samples with the highest outlier scores are the faulty samples. The experimental results with five state-of-the-art algorithms on publicly available datasets show that the GNNBFD algorithm improves the AUC by 6.4% compared to the next best algorithm, proving that the GNNBFD algorithm is effective and feasible.

## Introduction

With the rapid development of science and technology, mechanical equipment is widely used in modern industry. And the health monitoring technology of mechanical systems is also an essential issue in modern industry^1^. Rotating machinery is frequently used in industrial machinery and equipment, and its condition detection and fault diagnosis are of great significance in ensuring the reliability and safety of machinery in modern industrial systems^2^. Rolling bearing is a crucial component of rotating machinery. Due to its particular working environment, rolling bearing is prone to accidental failure and damage under high speed and heavy load as well as repeated high-temperature con-tact, which will directly affect the whole machine's performance, thus leading to serious safety hazards and high maintenance costs^3^. Bearing failure is the most common type of failure in rotating machinery systems, and according to statistics, 30–40% of rotating machinery failures are caused by bearing defects^4^. Therefore, efficiently intelligent fault diagnosis techniques for rolling bearings have been a vital research element in mechanical failures in the past decades.

Bearing fault diagnosis technology has undergone three stages: manual experience, signal processing, and intelligence^5^. Traditional mechanical fault diagnosis methods and theories can play a good role for simple systems with a single process, single fault, and gradual fault, but for multi-process, multi-fault, and sudden fault, as well as complex, large, highly automated large equipment and systems have more significant limitations^6^. Nowadays, with the development of sensors and computer systems, the amount of data describing the status of mechanical equipment has increased exponentially. Artificial intelligence methods can extract hidden fault features from these large-scale data sets and learn new fault types, significantly improving fault diagnosis accuracy while reducing the labor cost and diagnostic uncertainty of traditional methods^7–10^. However, the deep learning-based method considers the objects to be independently distributed and cannot take into account the correlation between objects during the training process, so it is still difficult to identify some of the early fault signals. In this paper, we propose a graph neural network-based bearing fault detection method in order to improve the accuracy of bearing fault detection.

Our main contributions are summarized as follows:

1. We convert the time-series signal of vibration into non-Euclidean structured graph data by methods such as feature transformation and similarity measurement.
2. A method for extracting features of vibration signals using graph neural networks is proposed. By feeding the vibration signal with the constructed graph into the graph neural network for training, the object after the training is completed can contain a wider range of neighborhood information.
3. In order to improve the usability of the algorithm in the real world, an ensemble learning approach is proposed to improve the robustness of the proposed algorithm.

## Related work

Up to now, a lot of research has been conducted on the intelligent diagnosis of bearing faults. Early widely used machine learning algorithms, such as PCA (Principal Component Analysis), SVM (Support Vector Machine), KNN (K-Nearest Neighbor), etc., have achieved satisfactory results in intelligent diagnosis, and the classification accuracy has improved significantly compared with the traditional methods. However, classical machine learning algorithms cannot learn nonlinear relationships^11^, and it isn't easy to find suitable shallow machine learning methods when there are highly complex and difficult to understand nonlinear relationships between input data (samples) and output data (labels). As a branch of machine learning, deep learning is highly capable of modeling nonlinearities with high flexibility and performs much better in dealing with realistic and complex problems. Therefore, deep learning has been introduced into bearing fault diagnosis to obtain a higher correct rate of fault diagnosis in complex environments^12,13^.

Many deep learning-based bearing fault diagnosis algorithms have been proposed as deep learning evolves.

CNN (Convolutional Neural Networks) is the most representative model of deep learning. Janssens et al.^14^ was the first paper to apply convolutional neural networks to bearing fault diagnosis, using the spatial structure in the data to effectively capture the covariance of the frequency decomposition of accelerometer signals; to balance the training speed and accuracy of the model, Guo et al.^15^ improved the traditional convolutional neural networks model by adding adaptive learning rate and momentum components to the weight update process; Xia et al.^16^ in the training process of convolutional neural networks both temporal and spatial information of the raw data from multiple sensors are considered; To deal with mechanical vibration signals with variable sequence length, Zhang et al.^17^ proposed a bearing fault diagnosis method based on residual learning algorithm, and the whole network uses a 1-dimensional convolutional layer to obtain local sequence features of the data information stream; For data that are difficult to obtain labels in practical situations, Meng et al.^18^ proposed a data enhancement technique, using deep convolutional neural network with residual learning algorithm as the main structure to obtain higher diagnostic accuracy with limited training data; Zhang et al.^19^ used a deep full convolutional neural network (DFCNN) containing four pairs of convolutional pooling layer pairs to convert vibration signals into images as input; Choudhary et al.^8^ proposed a fault diagnosis method for rotating machinery bearings combining CNN and thermal images, using various fault conditions explored the availability of thermal imaging in bearing fault diagnosis; Xu et al.^20^ proposed a rolling bearing fault diagnosis model based on online transfer convolutional neural network (OTCNN) with pre-trained network model and source domain features.

AE (Autoencoder) is an unsupervised approach to deep learning. In^21^, the maximum correlation entropy was used as the loss function of the deep autoencoder and the critical parameters of the deep autoencoder were optimized to fit the signal characteristics using an artificial fish swarm algorithm; Wang et al.^22^ used a Gaussian radial basis kernel function and acoustic emission method for fault diagnosis of bearings with high diagnostic accuracy and applicability; Shao et al.^23^ proposed an ensemble deep autoencoder for intelligent fault diagnosis of rolling bearings (EDAEs) method for unsupervised feature learning from measured vibration signals; similar to^24–26^ also improved on SAEs (Stacked Autoencoders) for fault diagnosis of bearings, both with improved detection results compared to traditional SAEs; Zhang et al.^27^ proposed a semi-supervised learning method based on a depth generating model of variational autoencoder (VAE), The VAE generation function is used to improve the classification performance when only a tiny portion of the data has labels; Cui et al.^28^ proposed a rolling bearing fault detection and classification method combining feature distance stacked autoencoder (FD-SAE) and support vector machines by organically combining machine learning and deep learning methods; Shao et al.^29^ used Morlet wavelet activation function to establish an accurate non-smooth vibration data based on stacked autoencoder with an accurate nonlinear mapping between the original non-stationary vibration data and various fault states using Morlet wavelet activation function; Ma et al.^30^ applied the weak magnetic detection method to rolling bearing whole life cycle monitoring with an improved variational autoencoder; Li et al.^31^ proposed a unified framework combining predictive generative denoising autoencoder (PGDAE) and deep coral network (DCN).

DBN (Deep Belief Network) is a simple combination of unsupervised networks. Chen and Li^32^ first applied deep belief network to bearing fault diagnosis and proposed a multi-sensor feature fusion diagnosis method for bearing faults based on stacked autoencoder and deep belief network; Hoang et al.^33^ automatically extracted bearing fault features from signals by DBN and then used Dempster-Shafer evidence theory combined with information from different sensors to predict bearing fault types; Liang et al.^34^ implemented a four-layer DBN that processes sensor data through multiple DBNs for feature extraction; Xu et al.^35^ combined clustering model affinity propagation (AP) with a DBN containing multiple hidden layers for fault diagnosis; Yu et al.^36^ combined maximum overlap discrete wavelet packet transform (MODWPT) and deep belief network methods to analyze rolling bearing fault features and identify fault states; Zhu et al.^37^ used principal component analysis to extract fault features and then used DBN for bearing fault diagnosis; Gao et al.^38^ focused on the structure and momentum of neural networks and used summary optimization algorithm to optimize the network structure of DBN; Niu et al.^39^ used particle swarm optimization (PSO) and adaptive training strategy to improve DBN to achieve higher accuracy and faster convergence speed.

In addition to CNN, AE and DBN, some common deep learning methods have also been applied to bearing fault diagnosis. For example^40–42^, used generative adversarial networks and their variants for bearing fault diagnosis; with the birth of LSTM (Long Short Term Memory network), References^43–45^ improved RNN (Recurrent Neural Network) and applied it to bearing fault diagnosis; and Refs.^46,47^ proposed a bearing fault diagnosis method with higher diagnostic accuracy based on reinforcement learning. In recent years, researchers have borrowed ideas from convolutional networks, recurrent networks, and deep autoencoders to define and design neural network structures for processing graph data, and graph neural networks have come into being, but up to now, there is almost no research related to the application of graph neural networks to bearing fault diagnosis.

## Model

**Figure 1:**
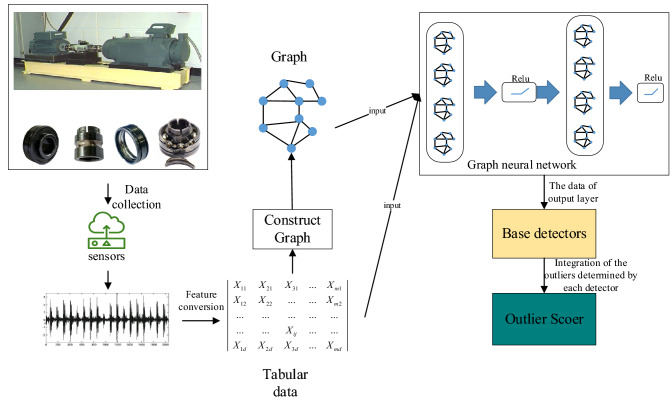
Flow chart of bearing fault detection based on graph neural network.

Among the collected bearing vibration signals, there are normal vibration signals and faulty vibration signals. The vibration signals are converted into nodes in the graph by means of data slicing and feature transformation. This converts the fault detection of bearings into a node classification task in machine learning. For the problem that early bearing fault signals are weak and difficult to distinguish from normal signals, we propose a graph neural network-based bearing fault detection method (GNNBFD). This method contains five main parts, which are: (1) dataset process, (2) construct graph, (3) graph neural network, (4) ensemble, and (5) outlier score. In this section, we will describe each part of the method in detail. Figure 1 represents the detection process of GNNBFD algorithm.

### Dataset process

**Figure 2:**
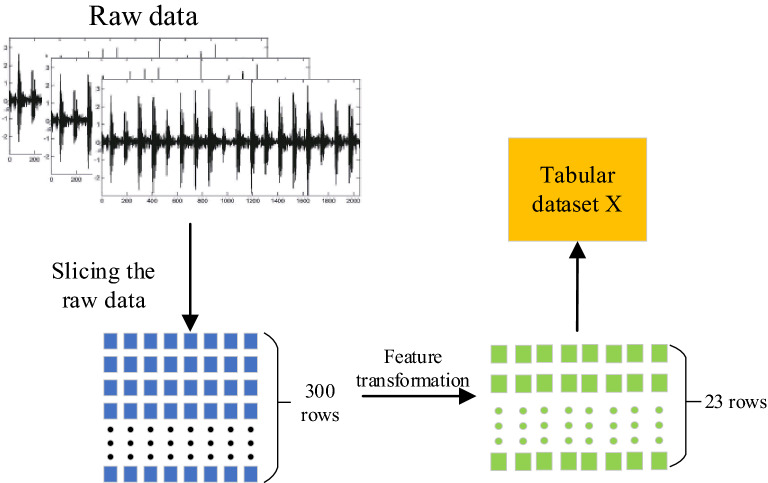
Flow chart of data process.

Since it is more difficult for neural networks to extract features from the original dataset, the dataset needs to be processed first to improve the detection accuracy of the algorithm. The processing of the dataset consists of two main steps: (1) slicing the dataset; (2) feature transformation. The flow chart of dataset process are shown in Fig. 2.

#### Slice and dice the dataset

The data set for bearing fault detection is usually N*1 of time-series data, and the data set is sliced in a segment of 300 data points, after which the original data is transformed into a matrix of 300*(*N*/300). The transformed data set contains a total of *N*/300 subsamples, and each subsample consists of 300 data points. It is worth noting that our slicing method for the dataset is obtained using a non-overlapping moving window.

#### Feature transformation

For each subsample, 23 features in the time and frequency domains are calculated and used as input to the subsequent model.

Based on Table 1, the index is calculated for each sample. Four steps are required.

1. Nine time domain indexes are calculated as follows:
  1 $$ I{ = }\left[ {I_{1} ,I_{2} ,I_{3} ,I_{4} ,I_{5} ,I_{6} ,I_{7} ,I_{8} ,I_{9} } \right] $$
2. *E*_WPD_ is obtained by calculating WPD energy (parameters* j* = 3 and wavelet Db20).
  2 $$ W_{WPD} { = }\left[ {E_{WPD}^{1} ,E_{WPD}^{2} ,E_{WPD}^{3} ,E_{WPD}^{4} ,E_{WPD}^{5} ,E_{WPD}^{6} ,E_{WPD}^{7} ,E_{WPD}^{8} } \right] $$
3. EEMD energy is calculated to obtain a dataset as follows:
  3 $$ W_{EEMD} { = }\left[ {E_{EEMD}^{1} ,E_{EEMD}^{2} ,E_{EEMD}^{3} ,E_{EEMD}^{4} ,E_{EEMD}^{5} ,E_{EEMD}^{6} } \right] $$
4. *I*, *W*_*WPD*_, *W*_*EEMD*_ are combined into a dataset as follows:
  4 $$ X = \left[ {I,W_{WPD} ,W_{EEMD} } \right] $$

**Table 1:** Indexes and the calculation formulas.

Indexes	Formulas
1. Standard deviation	$I_{1} = \sqrt {\sum\nolimits_{n = 1}^{N} {(x(n) - \overline{x})}^{2} /N}$
2. Peak	$I_{2} = \max |x(n)|$
3. Skewness	$I_{3} = \sum\nolimits_{n = 1}^{N} {(x(n) - \overline{x})^{3} } /(N - 1)I_{1}^{3}$
4. Kurtosis	$I_{4} = \sum\nolimits_{n = 1}^{N} {(x(n) - \overline{x})^{4} } /(N - 1)I_{1}^{4}$
5. Root mean square	$I_{5} = \sqrt {\sum\nolimits_{n = 1}^{N} {x(n)}^{2} /N}$
6. Crest factor	$I_{6} = I_{2} /\sqrt {\sum\nolimits_{n = 1}^{N} {x(n)}^{2} /N}$
7. Square	$I_{7} = I_{2} /\left( {\sum\nolimits_{n = 1}^{N} {\sqrt {x(n)} } /N} \right)^{2}$
8. Shape factor	$I_{8} = \sqrt {N\sum\nolimits_{n = 1}^{N} {x(n)^{2} } } /\sum\nolimits_{n = 1}^{N} {|x(n)|}$
9. Impulse factor	$I_{9} = \max |x(n)|/\left( {\sum\nolimits_{n = 1}^{N} {{|}x(n)} {|}/N} \right)$
10. WPD energy	$I_{10} = \sum\nolimits_{i = 1}^{N} {|x_{i} (n)|}^{2} /\sum\nolimits_{i = 0}^{{2^{j - 1} }} {\sum\nolimits_{n = 1}^{N} {|x_{i} (n)|} }^{2}$
11. EEMD energy	$I_{11} = \sum\nolimits_{n = 1}^{N} {|IMF_{i} (n)|}^{2} /\sum\nolimits_{i = 1}^{NI} {\sum\nolimits_{n = 1}^{N} {|IMF_{i} (n)|} }^{2}$

*x*(n)and $\overline{x}$ denote the data sequence and mean of the data sequence, respectively; *N* is the number of the data points. *x*_*i*_(*n*) is the decomposition coefficient sequence of the ith (*i* = 0, 1,…, 2^*j*^ − 1, *j* is the WPD decomposition level) frequency band using WPD; *IMF*_*i*_(*n*) is the *i*th data sequence after EEMD, and *NI* is the decomposition level using EEMD.

The feature transformation of the sliced dataset reduces the redundant information of the subsamples and can effectively reduce the computational effort of the subsequent model. The extracted 23 time-domain and frequency-domain features can adequately reflect the information contained in the samples and facilitate further processing of the subsequent model.

### Construct graph

Since the subsamples processed by Section “Dataset process” are independent of each other, there is no interconnectivity between the subsamples. Traditional deep learning methods would input the subsamples directly into the model for training, but this method does not consider the correlation between the subsamples. For this reason, we propose a method for constructing correlations between subsamples, called construct graph.

**Figure 3:**
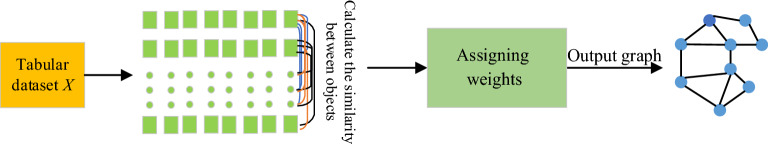
Flow chart of construct graph.

Construct graph mainly considers the similarity between subsamples, and the higher the similarity, the greater the weight of the connection between them. Construct graph consists of (1) calculating the similarity between subsamples, (2) assigning weights, and (3) outputting the graph. The flow chart of construct graph are show in Fig. 3.

#### Similarity

Let *X* = {*X*_1_, *X*_2_, *X*_3_…, *X*_*m*_} indicate the processed dataset, where *X*_*i*_ ≤ *X*_*i*1_, *X*_*i*2_, *X*_*i*3_…. *X*_*id*_ > indicates a subsamples in *X*, and *d* indicates the dimension of *X*. *X*_*id*_ indicates the value of subsamples *X*_*i*_ in the *d*th dimension.

We use the normalized Euclidean distance as a measure of similarity between subsamples. *Dist* (*x*_*i*_, *x*_*j*_) represents the similarity between subsamples *x*_*i*_ and *x*_*j*_ and is calculated as shown in the following equation:

5 $$ Dist\left( {x_{i} ,x_{j} } \right) = (x_{i} - x_{j} )V^{{ - {1}}} (x_{i} - x_{j} )\prime $$

where *V* is the *n*-by *n* diagonal matrix whose *j*th diagonal element is *x*_*i*_^2^, where *x*_*j*_ is a vector of scaling factors for each dimension. When the value between *Dist* (*x*_*i*_, *x*_*j*_) is larger, it means that the similarity between two objects is higher.

#### Assigning weights

First, the top *k* subsamples with the highest similarity to *x*_*i*_ subsamples are selected, and they form the set of neighbors of *x*_*i*_, which is denoted as *N*_*k*_ (*x*_*i*_). Then calculate the weights between these *k* subsamples and *x*_*i*_, which are calculated as shown in the following equation.

6 $$ W(X_{i} ,X_{j} ) = \left\{ \begin{gathered} \frac{{Dist(X_{i} ,X_{j} )}}{{\sum\limits_{j = 1}^{k} {Dist(X_{i} ,X_{j} )} }} \, ,X_{j} \in N_{k} (X_{i} ) \hfill \\ 0 \, ,X_{j} \notin N_{k} (X_{i} ) \hfill \\ \end{gathered} \right. $$

#### Output graph

By calculating the similarity and assigning weights in the first two steps, we can connect the subsamples to each other. If the weights between subsamples are greater than 0, there is a connected edge between them; otherwise there is no connection between them. The subsamples are connected to each other to form the graph, and we represent the constructed graph by the adjacency matrix *A*.

$$ A{ = } \left | \begin{array}{*{20}c}  & 1 & {W(X_{{2}} ,X_{1} )} & {...} & {...} &{W(X_{m} ,X_{1} )}  & \\  & {W(X_{{1}} ,X_{{2}} )} & 1 & {...} & {...} & {W(X_{m} ,X_{2} )}  & \\ & {...} & {...} & {...} & {...} & {...}  & \\  & {...} & {...} & {W(X_{i} ,X_{j} )} & {...} & {...}  & \\  & {W(X_{1} ,X_{m} )} & {W(X_{2} ,X_{m} )} &  {...} &  {...} &  1  & \\ \end{array} \right |$$

The values of the diagonal elements in the adjacency matrix *A* are all 1, which indicates that the subsample itself is connected to itself. In this way, the feature information of the subsample itself can be effectively prevented from being lost during the training process of the subsequent model.

### Graph neural network

Traditional neural network structures such as convolutional neural networks, recurrent neural network, etc. receive data in Euclidean space as input, and they cannot handle data structures in non-Euclidean space, such as graphs. Therefore, we will use graph neural networks to handle graph data. GNN a framework to learn directly from graph structured data using deep learning.

We use a recurrent neural structure to propagate the neighbor information until reach a stable immobility point to learn the representation of the target node, that is facilitate the subsequent fault detection task. After feature extraction by the graph neural network, each graph node contains not only its own information, but also the feature information of its neighbors. Our forward model then takes the simple form:

7 $$ Z = f(X,A) = ReLU((ReLU(XAW^{(0)} - b^{(0)} )AW^{(1)} - b^{(1)} ) $$

Here, $W \in R^{D \times H}$ is an input-to-hidden weight matrix for a hidden layer with *H* feature maps, and $b \in R^{H}$ is an input-to-hidden biases matrix. The GNN weights *W*^(0)^, *W*^(1)^ and biases *b*^(0)^, *b*^(1)^ are trained using the gradient descent.

**Figure 4:**
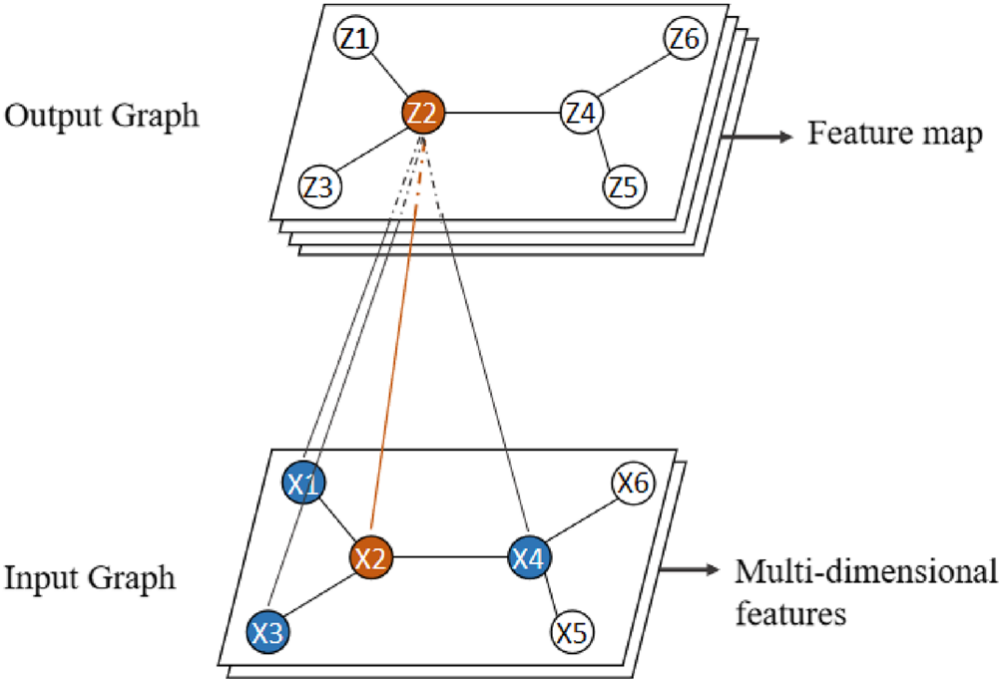
The structure of GNN.


As shown in Fig. 4, the GNN model is based on an information propagation mechanism, where each node exchanges information (propagates) with other nodes through continuous iterative updates to reach a stable state. When the information flow of the whole graph smooth’s out, each node has information about itself and its neighboring nodes.

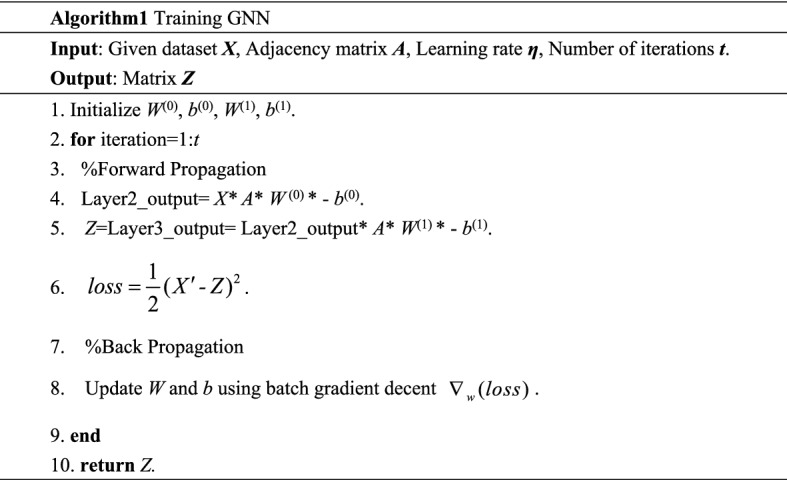


### Ensemble

In this paper, we combine the detection of Z-matrices using multiple outlier detection algorithms, a process called ensemble. Each subsample of the Z matrix output by the GNN network contains not only its own eigenvalues, but also those of its neighbors. Inputting the Z-matrix into the outlier detection algorithm for detection helps to maximize the separation of normal objects from outliers in the dataset.

We use three classical outlier detection algorithms, LOF (Local Outlier Factor), KNN (K-Nearest Neighbor), and AE (Autoencoder), to detect the Z matrix. LOF is the classical density-based outlier detection algorithm, KNN is the distance-based outlier detection algorithm, and AE is the neural network-based outlier detection algorithm. Different types of outlier detection algorithms focus on detecting outliers with different distributions. Combining the above three types of algorithms can maximize the robustness of GNNBFD. Meanwhile, LOF, KNN, and AE are all unsupervised outlier detection algorithms, and the first two do not require training process. When training the AE algorithm, we set its learning rate to 0.0001, the hidden layer depth of the network to three layers, and the Adam optimizer is used for back propagation.

Each algorithm eventually outputs an outlier value for each subsample in the Z matrix. The outlier value indicates the probability that the subsample is a faulty sample, and the higher the outlier value, the more likely the sample is a faulty sample, and vice versa.

### Outlier score (OS)

We define the outlier value of the *i*th subsample as Outlier Score (*OS*), *OS* is calculated as shown in the following equation:

8 $$ OS_{i} = \frac{{\sum\limits_{j = 1}^{m} {OF_{j} } }}{m} $$

In the above equation, *OF*_*j*_ denotes the outlier factor assigned to the *i*th subsample by the *j*th outlier detection algorithm. *OS*_*i*_ denotes the average outlier score of the *i*th subsample over the *m* outlier detection algorithms. By averaging the values in this way, the misclassification rate of the algorithm is reduced and the reliability and robustness of the final detection results of the algorithm are improved.
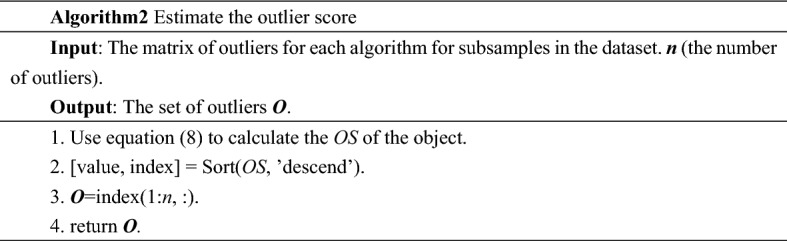


## Experiments

In this section, we will demonstrate the effectiveness of the proposed algorithm for bearing fault detection in detail in four parts. These four sections are: (a) introduction to the experimental environment and data set; (b) evaluation method and comparison algorithm; (c) experimental results and (d) effectiveness analysis.

In (a) Introduction to the experimental environment and dataset, we describe in detail the experimental platform, hardware and software configurations, and the introduction and processing of the dataset; in (b) valuation method and comparison algorithm section, we use four evaluation metrics to measure the performance of each algorithm in a comprehensive manner. In (c) Experimental results, we report the final detection results of the algorithms in detail and analyze the reasons for them. In (d) effectiveness analysis, we verified the effectiveness of the GNNBFD algorithm using k-fold cross-validation.

### Experimental environment and dataset

The hardware environment for the experiments is an Intel(R) Core(TM) i7-7700 3.60 GHz CPU with 8 GB of RAM. The software side contains the platform and operating system required for the experiments, and we implemented the code required for the model using Matlab 2020, and the operating system is Windows 10 Professional.

**Figure 5:**
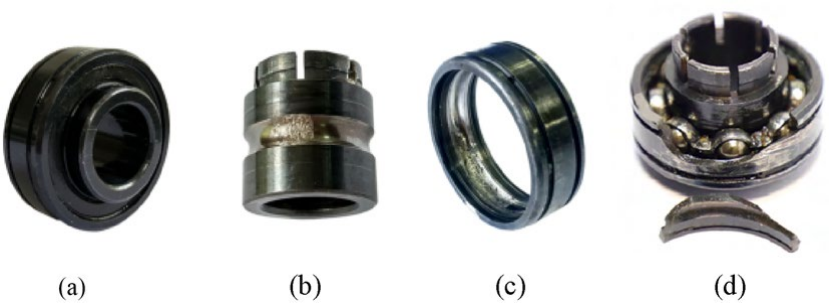
Normal and fault state of bearings (**a**) normal (**b**) inner race fault (**c**) outer race fault (**d**) ball fault.

The dataset is the publicly available Case Western Reserve University (CWRU) dataset. The advantage of using a publicly available dataset is that it is easy for other researchers to reproduce our experimental results. The bearing type is SKF6250, bearing location is Drive-end, Sample frequency (Hz) is 12000 Hz and the motor speed (rpm) is 1797. We divided the collected experimental data into 3 groups, each group contains 4 states, which are: (1) 0.1778 mm inner race fault, 0.3556 mm inner race fault, 0.5334 mm inner race fault and normal base; (2) 0.1778 mm ball fault, 0.3556 mm ball fault, 0.5334 mm ball fault and normal base; (3) 0.1778 mm outer race fault, 0.3556 mm outer race fault, 0.5334 mm outer race fault and normal base. Figure 5 shows the normal bearing and the three faulty bearing states.

Taking the first set of inner circle faults as an example, the normal sample contains a total of 240,000 data points and the faulty sample contains 120,000 data points. We divide the normal sample and the faulty sample into a sub-sample with 300 data points. Among the divided fault samples, 20 samples are randomly selected as outliers and form the first data set together with the normal objects. They were processed with the two data sets according to the same method, and thus three data sets were obtained, and the details of the data sets are summarized in Table 2.

**Table 2:** Summary of datasets.

Group 1			Group 2			Group 3		
Fault type	Fault diameter (mm)	Sample number	Fault type	Fault diameter (mm)	Sample number	Fault type	Fault diameter (mm)	Sample number
Normal	0	800	Normal	0	800	Normal	0	800
Inner race fault	0.1778	20	Ball fault	0.1778	20	Outer race fault	0.1778	20
	0.3556	20		0.3556	20		0.3556	20
	0.5334	20		0.5334	20		0.5334	20

### Evaluation methods and comparison algorithms

We use the receiver operating characteristic (ROC) curve and corresponding area under the curve (AUC), accuracy (ACC), detection rate (DR), and false alarm rate (FAR) to measure the detection performance. Higher AUC, ACC, and DR values and lower FAR indicate better performance.

The calculation method of ACC, DR, FAR can be obtained from the confusion matrix in Table 3.

9 $$ ACC = \frac{TP + TN}{{TP + TN + FP + FN}} $$

10 $$ DR = \frac{TP}{{TP + FN}} $$

11 $$ FAR = \frac{FP}{{TN + FP}} $$

**Table 3:** Confusion matrix.

True class	Predicted class	
	Positive	Negative
Positive	True positive (TP)	False negative (FN)
Negative	False positive (FP)	True negative (TN)

We compare GNNBFD with five representative outlier detection algorithms. These algorithms are common types in the outlier detection field, and they are used as comparison algorithms in most of the related literature. They can be divided into five categories: (i) Neuron network-based, SO-GAAL (Single Objective Generative Adversarial Active Learning); (ii) Graph-based, CutPC (graph-based clustering method using noise cutting); (iii) Local outlier factor-based, LOF; (iv) Distance-based, KNN; (v) Isolation-based, IForest. The parameter settings are summarized in Table 4.

**Table 4:** Parameter setting.

Algorithms	*k* (number of nearest neighbors)	Learning rate	Number of iterations	Number of layers	*xi* (relative decrease in density)	minpts (number of points required to form a cluster)	Number of isolation trees and subsample size
GNNBFD	2–100	0.0001–0.002	10–100	3	–	–	–
SO-GAAL	–	0.0001–0.002	10–100	3	–	–	–
CutPC	–	–	–	–	–	–	–
LOF	2–100	–	–	–	–	–	–
KNN	2–100	–	–	–	–	–	–
IForest	–	–	–	–	–	–	100–256

### Experiment results

**Figure 6:**
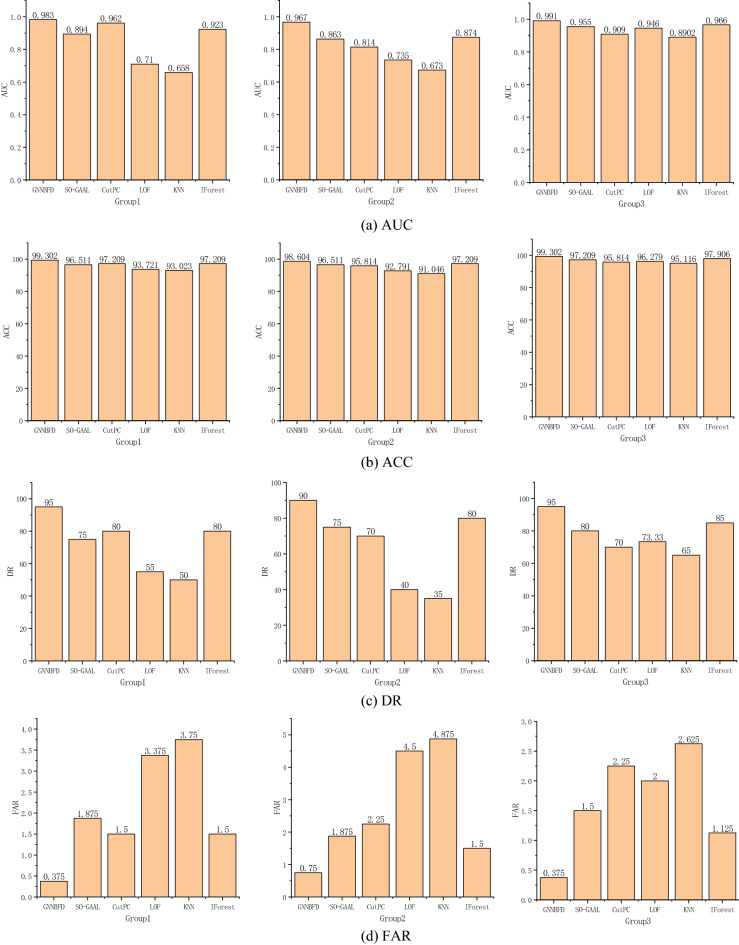
Experimental results.

Experimental results on real-world datasets are shown in Fig. 6. We adjust the parameters 10 different times for all algorithms and choose the best result among the 10 times as the evaluation of the final performance of the algorithm.

Observing the experimental results, some interesting information can be found.

1. Among the four evaluation methods, the GNNBFD achieves the best results. Compared with the next best IForest algorithm, GNNBFD improves the AUC value by 6.4% on average, which proves the validity of the proposed algorithm.
2. In the Group2 dataset, most algorithms have poor detection results. This is due to the weak fault vibration signal of the ball and the low degree of deviation of the normal signal from the fault signal. Since the GNNBFD method can learn the broader neighborhood information of the object, which enables the fault signal with low degree of deviation to produce a greater degree of deviation after the graph neural network embedding.
3. In the process of converting the vibration signals into graphs, connectivity relations are constructed for the objects. After training with the graph neural network, the low-dimensional embedding of the objects contains more valuable information and changes the distribution pattern from the original one. Therefore, the base detector is able to detect the faulty and normal objects more accurately when performing detection.

The experimental results prove that the GNNBFD is effective and feasible.

### Effectiveness analysis

K-fold cross-validation is a commonly used technique for evaluating the performance of machine learning models. By using K-fold cross-validation, a more accurate estimate of a model's performance can be obtained. Furthermore, K-fold cross-validation can help detect whether a model is over fitting, as it enables evaluation of the model on a larger amount of data.

To analyze the effectiveness of the GNNBFD algorithm proposed in this paper, we performed K-fold cross-validation. Specifically, we performed a fivefold cross-validation on each of the three datasets. Each time, 150 normal objects and 30 faulty objects were selected from each dataset, and the performance of the proposed algorithm was measured by the AUC value. The experimental results are shown in Table 5.

**Table 5:** K-fold cross validation.

k-fold	Group 1	Group 2	Group 3
1	0.986	0.971	0.994
2	0.982	0.977	0.993
3	0.974	0.982	0.988
4	0.991	0.963	0.989
5	0.987	0.981	0.991
Avg	0.984	0.975	0.991

To ensure that when performing fault detection, GNNBFD is blind to the samples. We used random sampling and randomly sorted these samples. Observing the experimental results in Table 5, it can be seen that the GNNBFD algorithm still has good detection results with a small number of samples. The average AUC values in the three datasets are 0.984, 0.975 and 0.991, it proves that the proposed algorithm can effectively detect the bearing fault signals.

## Conclusion

In this paper, a graph neural network-based bearing fault detection method is proposed to improve the accuracy of bearing fault detection. The graph neural network has a very powerful feature mapping capability, which can fit the feature values of the sample and its neighbors simultaneously. The samples outputted by the GNN network after mapping can be more easily separated from normal samples and fault samples by the base detector algorithm. Considering the higher requirements for algorithm robustness in real production environments, we use an integrated technique to synthesize the detection results of the base detector to make the GNNBFD algorithm more stable and efficient. Experiments on publicly available datasets show that the GNNBFD algorithm can successfully detect most of the fault samples in the dataset. In the future work, we will mainly study how to improve the detection performance of the GNNBFD with a deeper network structure.

## Data Availability

The datasets generated and/or analyzed during the current study are available in the Case Western Reserve University (CWRU) repository, https://github.com/yyxyz/CaseWesternReserveUniversityData.
